# Microbial protection favors parasite tolerance and alters host-parasite coevolutionary dynamics

**DOI:** 10.1016/j.cub.2022.01.063

**Published:** 2022-04-11

**Authors:** Charlotte Rafaluk-Mohr, Michael Gerth, Jordan E. Sealey, Alice K.E. Ekroth, Aziz A. Aboobaker, Anke Kloock, Kayla C. King

**Affiliations:** 1 Department of Zoology, University of Oxford, 11a Mansfield Road, Oxford OX1 3SZ, UK; 2 Department of Biological and Medical Sciences, Oxford Brookes University, Gipsy Lane, Headington, Oxford OX3 0BP, UK

**Keywords:** host-parasite coevolution, protective symbiosis, microbiota, Red Queen, Arms Race, tolerance, diversity, recombination

## Abstract

Coevolution between hosts and parasites is a major driver of rapid evolutionary change^1^ and diversification.^2^^,^^3^ However, direct antagonistic interactions between hosts and parasites could be disrupted^4^ when host microbiota form a line of defense, a phenomenon widespread across animal and plant species.^5^^,^^6^ By suppressing parasite infection, protective microbiota could reduce the need for host-based defenses and favor host support for microbiota colonization,^6^ raising the possibility that the microbiota can alter host-parasite coevolutionary patterns and processes.^7^ Here, using an experimental evolution approach, we co-passaged populations of nematode host (*Caenorhabditis elegans*) and parasites (*Staphylococcus aureus*) when hosts were colonized (or not) by protective bacteria (*Enterococcus faecalis*). We found that microbial protection during coevolution resulted in the evolution of host mortality tolerance—higher survival following parasite infection—and in parasites adapting to microbial defenses. Compared to unprotected host-parasite coevolution, the protected treatment was associated with reduced dominance of fluctuating selection dynamics in host populations. No differences in host recombination rate or genetic diversity were detected. Genomic divergence was observed between parasite populations coevolved in protected and unprotected hosts. These findings indicate that protective host microbiota can determine the evolution of host defense strategies and shape host-parasite coevolutionary dynamics.

## Highlights

• Microbial protection resulted in the evolution of host mortality tolerance
• Parasites adapted to counter microbial defenses within hosts
• Protective microbes reduced fluctuating selection dynamics
• Microbial protection did not impact host genetic diversity or recombination rates

## Teaser

Protective microbes are widespread across host species and likely play a key role in mediating host interactions with parasites. Rafaluk-Mohr et al. show that microbial protection against infection can influence the host defense strategies that evolve, as well as the patterns and processes of host-parasite coevolution.

## Results and discussion

**Figure 1:**
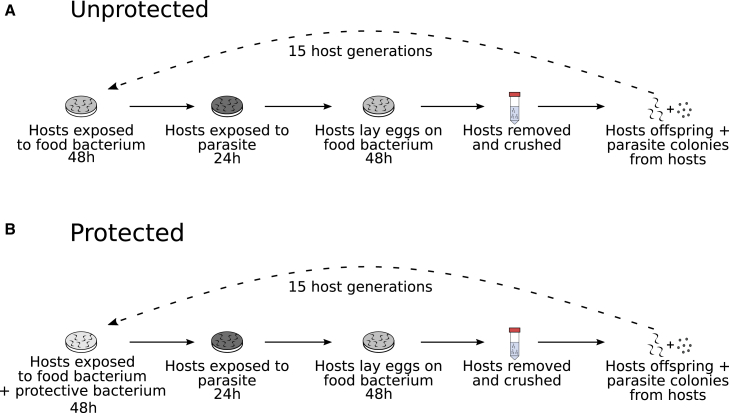
Design of the coevolution experiment (A) Unprotected and (B) protected treatments. Host, *Caenorhabditis elegans*; protective bacterium, *Enterococcus faecalis*; parasite, *Staphylococcus aureus*.

We experimentally co-evolved C. *elegans* worms with the virulent parasite *S. aureus* for 15 host generations while the hosts were (“protected” treatment) or were not (“unprotected” treatment) colonized by *E. faecalis* during development (Figure 1). *E. faecalis* can be mildly pathogenic to nematodes but has been observed in natural microbiomes to possess protective traits.^8^, ^9^, ^10^, ^11^ Moreover, although *C. elegans* likely encounters *Staphylococcus* species in its natural habitat,^12^^,^^13^ interactions with *S. aureus* here are novel. This parasite is ingested by the host and can kill when it lyses nematode gut wall cells.^14^ *E. faecalis* bacteria produce anti-microbial superoxides^15^ and steal parasite-produced siderophores,^16^ which strongly inhibit *S. aureus* infection. We tested for effects of this protective bacterium on the outcomes of host-parasite coevolution and the dynamics of allele frequency change.

**Figure 2:**
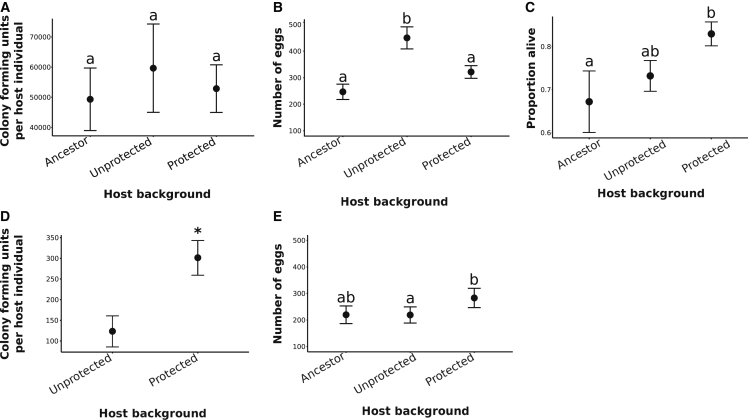
Host adaptation during unprotected and protected coevolution Evolution of host tolerance strategies against parasite infection. (A) Colony-forming units (CFUs) of ancestral parasites per host across ancestral, unprotected coevolved, and protected coevolved backgrounds (n = 58). Error bars are SEM. (B) Number of eggs per host across host backgrounds during infection with ancestral parasites (n = 52). Error bars are SEM. C) Proportion of live hosts across host backgrounds during ancestral parasite infection (n = 51). Error bars are SEM. (D) CFUs of protective bacteria per host across host backgrounds during ancestral parasite infection (n = 51). Error bars are SEM. (E) Number of eggs from protected hosts across host backgrounds during ancestral parasite infection (n = 20). Error bars are SEM.

As a consequence of coevolution with parasites, hosts may evolve resistance to infection, tolerance, or a combination of both defense strategies.^17^^,^^18^ Resistant hosts clear or reduce parasite loads, whereas tolerant hosts maintain their fitness despite infection.^17^^,^^18^ Tolerance can be further divided into fecundity and mortality tolerance, depending on whether hosts buffer fitness by maintaining reproductive output or survival, respectively.^18^ After 15 host generations of protected or unprotected coevolution with parasites, different host tolerance strategies emerged between treatments. In both treatments, hosts evolved to tolerate rather than clear the infection (Figure 2A). Unprotected hosts evolved “fecundity tolerance”: egg production increased post-infection compared to the protected treatment and ancestor (nested linear model: chi-square = 8.34, d.f. = 2, p = 0.015; Figure 2B), enhancing their fecundity compensation response.^19^ Conversely, protected hosts evolved “mortality tolerance,” with enhanced survival following infection, even when microbe-mediated defenses were removed (quasibinomial general linearized model: chi-square = 9.63, d.f. = 2, p = 0.008; Figure 2C). Host mortality tolerance has been predicted to evolve in response to less virulent parasites because initial costs of infection are lower, particularly in comparison to mounting an immune response.^18^^,^^20^ Through reducing the virulence of parasite infection, the presence of protective microbes may have driven the evolution of host mortality tolerance.

We hypothesized that protected hosts would evolve to maximize anti-infective benefits by supporting increased colonization by protective microbes. This positive relationship between number of protective bacteria cells and the strength of protection has been found previously in this system^15^ and other symbioses^21^. We exposed hosts from each treatment to static *E. faecalis* and found that protected treatment hosts accumulated higher loads of protective bacteria than unprotected hosts following our experimental evolution regime (nested linear model: chi-square = 13.5, d.f. = 1, p < 0.001; Figure 2D). These hosts were better defended and had more offspring (generalized linear model: chi-square = 5.67, d.f. = 2, p = 0.05; Figure 2E). Hosts may have survived parasite infection by preventing internal damage. A lower proportion of worms from the protected coevolution treatment leaked artificially colored food bacteria through their guts^22^ following infection (55%), compared to those in the unprotected coevolution treatment (60%) or ancestral worms (64%). This outcome was supported by gene ontology (GO) term enrichment analysis on SNPs under selection following whole-genome sequencing of coevolved worm populations. Terms associated with cell membrane integrity were significantly over-represented in the protected treatment (Panther slim over-representation test; Tables S1, S2, and S1). These results suggest that protected hosts might tolerate infection by preventing parasite lysis of gut cells.

**Figure 3:**
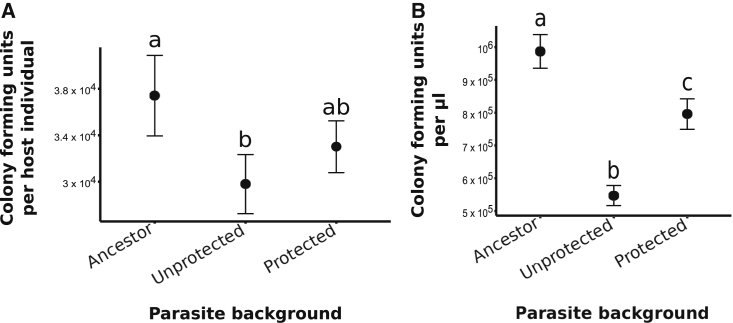
Parasite adaptation during unprotected and protected coevolution (A) CFUs of parasites per protected host for each parasite background across pooled host backgrounds (n = 265). Error bars are SEM. (B) CFUs of parasites per μL media grown in *in vitro* co-culture with protective bacteria (n = 96). Error bars are SEM.

Parasites from the protected treatment were reciprocally impacted during coevolution. Parasites were found to have adapted to the experimental setup as, across both coevolution treatments, their growth alongside the protective bacterium was lower than for the ancestor (Figure 3A). Nevertheless, parasite growth in the presence of protective bacteria *in vivo* (nested linear model: chi-square = 8.19, d.f. = 2, p = 0.017; Figure 3A) and *in vitro* (nested linear model: chi-square = 40.74, d.f. = 2, p < 0.001; Figure 3B) was significantly higher in the protected compared to the unprotected treatment. These shared *in vitro* and *in vivo* findings reveal that parasites adapted to direct antagonism from protective microbes, and this was not an effect mitigated by the host.

Coevolutionary dynamics are often described as either directional (Arms Race) or fluctuating (Red Queen). The type depends on whether there is reciprocal directional selection for host defenses and parasite infectivity^23^ or negative frequency-dependent selection causing fluctuations in allele frequencies.^24^ Using a population genomics approach, we examined whether host microbiota caused shifts in the mode of host-parasite coevolution. We sequenced entire host and parasite populations at multiple time points (ancestor, passage 8, passage 15) across both treatments. We then examined the type of coevolution occurring in each community by comparing the changes in frequency of each allele across time. For each host allele, selection coefficients were calculated using a series of binomial generalized linear models within each treatment and for the first (G0 to G8) and second (G8 to G15) halves of the experiment.^25^ If change was statistically significant in either half, the allele was considered to be under selection in that treatment. Consistent increases or decreases in allele frequency throughout both halves (+/− signs the same) marked an allele as under directional selection, but a change in the direction of frequency (+/− signs differed) indicated fluctuating selection.

**Figure 4:**
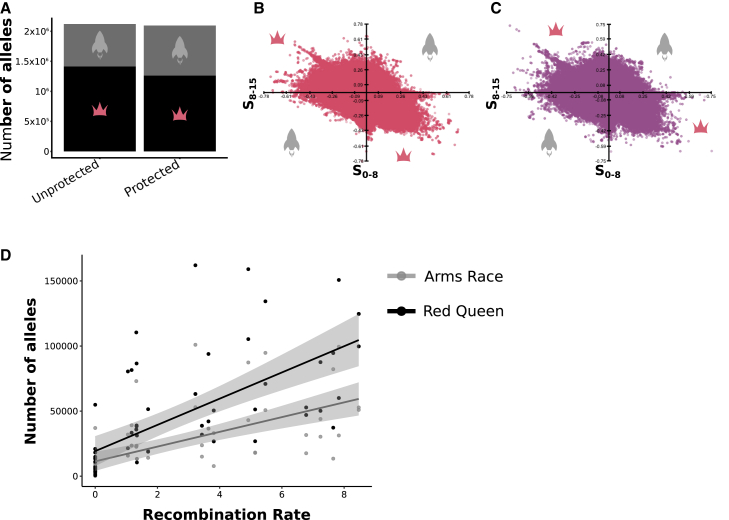
Host genomic evolution during unprotected and protected coevolution (A) Number of alleles under fluctuating (Red Queen) (dark gray bars) or directional (Arms Race) (pale gray bars) selection. (B) Selection coefficients for allele frequency changes in the host for the second (x) and first (y) half of the evolution experiment for the protected treatment. (C) Selection coefficients for allele frequency changes in the host for the second (x) and first (y) half of the evolution experiment for the unprotected treatment. (D) The relationship between recombination rate for a given chromosomal region^26^ and the number of alleles under selection for alleles under directional (Arms Race) or fluctuating (Red Queen) selection. Reciprocal analysis for the parasite can be found in Figures S1–S4 and Tables S1, S2, and S3.

We found that multiple modes of selection acting on hosts (Figure 4) and parasites (Figure S1) occurred during coevolution, in agreement with other studies tackling host-parasite coevolution at the genomic level.^25^^,^^27^^,^^28^ Fluctuating selection dominated in host populations across treatments—a greater proportion of host alleles fluctuated in frequency between time points. Due to the lower number of variants in the parasite treatment, clear differences in dynamics could not be detected. However, we did observe greater changes with more alleles going to fixation in the unprotected treatment (Figure S1). The prevalence of these dynamics nevertheless varied. Significantly more host alleles were under directional selection in the protected treatment (chi-square contingency table tests: chi-square = 35,542, d.f. = 1, p < 0.001; Figures 4A–4C). This shift away from fluctuating selection may be driven by the disruption of specific host gene-parasite gene interactions by protective bacteria, directional selection for host traits promoting colonization for protective microbiota, and the evolution of host mortality tolerance. Mortality tolerance is predicted to create positive evolutionary feedback between hosts and parasites, aligning their interests^18^^,^^29^ and in some cases leading to apparent commensalism.^30^^,^^31^ Extended host lifespan directly benefits parasite fitness because of more opportunities for infection.^17^^,^^18^ This reduction in antagonism could lead to new equilibria rather than open-ended cyclic dynamics^32^ and is thus consistent with less fluctuating selection on host populations we see here.

At the genomic level, we detected divergence between parasites coevolving with unprotected and protected host populations. On average, pairwise genetic distances of parasites between treatments were significantly different (supplemental information Figure S2; t = 2.6553, d.f. = 6.4692, p = 0.035). The targets of selection in the parasite populations were difficult to elucidate. The treatments did not differ overall in the parasite loci that acquired mutations or in the putative functions of genes under selection (Table S2; Figures S3 and S4).

Antagonistic coevolution with fluctuating selection dynamics is predicted to stably maintain population genetic diversity.^3^^,^^25^^,^^27^^,^^33^ We thus predicted that host diversity would be greatest during unprotected coevolution where we observed fluctuating selection to be more common. Host populations remained highly diverse throughout the experiment. There were 371,958 different allelic variants in the ancestral population with no significant loss over evolutionary time (343,474 in unprotected; 305,578 in protected; two-sample t test: t = 1.668, d.f. = 4.5556, p = 0.1619). More alleles under directional selection in host populations did not limit the impact of fluctuating selection on the maintenance of host diversity.

During coevolution via fluctuation selection dynamics, parasites have been shown to favor increased host recombination rates.^34^, ^35^, ^36^ The *C. elegans* population we used in this experiment was dioecious and obligately outcrossing,^37^ and we thus expected high levels of recombination in our experimental set up. The host genomic data revealed that alleles under different types of selection occurred in different regions of the chromosome. We hypothesized that this pattern was due to variable recombination rates along the chromosome. Using published recombination rates for *C. elegans*,^26^ we compared the number of alleles under selection for each treatment and selection mode across recombination rates. The recombination rates were measured as cM/Mb for each region of the chromosome, defined using segmented linear regressions.^26^ Across all treatments and modes of selection, we found a positive relationship between recombination rate and the number of alleles under selection (R^2^ = 0.36, d.f. = 1,118, p < 0.0001), but no difference between unprotected and protected treatments (F = 0.002, d.f. = 1, p = 0.96). That this relationship is similar between treatments supports the argument that host harm from infection is not critical for higher recombination rates to be advantageous.^38^ The mode of selection nevertheless had a significant impact on the strength of the correlation. The number of alleles under both directional (R^2^ = 0.37, d.f. = 1,58, p < 0.0001) and fluctuating selection (R^2^ = 0.43, d.f. = 1,58, p < 0.0001) showed a positive relationship with recombination rate and number of alleles under selection. However, the intercepts of the slopes for alleles under both modes of selection differed significantly (F = 4.7, d.f. = 1, p = 0.032), with a higher intercept for host alleles under fluctuating selection, suggesting a stronger relationship (Figure 4D). These results support previous work^34^, ^35^, ^36^ in highlighting that areas of the chromosome with high recombination rates can be under strong fluctuating selection.

Our study reveals that host microbiota plays a role in the ongoing evolution of infectious disease systems and can shape coevolution between hosts and parasites. Protection from resident microbes ultimately reduced the burden of parasite infection on hosts and selected for mortality tolerance as a defense strategy. Protective microbiota can defend hosts against a broad spectrum of enemy genotypes and species^39^^,^^40^ and even disrupt tight host-parasite genetic specificity in natural systems.^40^ Such specificity is generally assumed to underpin fluctuating coevolution,^41^ although coevolutionary cycles can occur in the absence of specificity.^42^ Here, the reduced dependence on these specific interactions for infection success dampened the dominance of fluctuating selection on hosts as well as the frequency changes in parasite alleles. The impacts of microbiota on host evolution are increasingly being uncovered.^43^, ^44^, ^45^, ^46^, ^47^ We should also consider the microbiota as a mediator of other environmental selection pressures faced by animals (e.g., thermal stress^48^) during their evolution. This focus could be particularly informative in studying the evolution of host defenses in the wild or lab systems where hosts possess their natural microbiota. The extent to which natural patterns of host tolerance of infection^49^ and host-parasite coevolution^50^ are more widely underpinned by protective microbiota remains to be tackled.

## STAR★Methods

### Key resources table

**Table:** 

REAGENT or RESOURCE	SOURCE	IDENTIFIER
**Deposited data**		

Whole genome resequencing data of evolved an ancestral host and parasite populationa	National Center for Biotechnology Information (NCBI)	BioProject: PRJNA673790
Phenotypic data	Mendeley Data	https://doi.org/10.17632/8tw4cfhzbc.1
Tryptic Soy Broth	Prepared by the media kitchen, Department of Biochemistry University of Oxford	N/A
Todd Hewitt Broth	Prepared by the media kitchen, Department of Biochemistry University of Oxford	N/A
Mannitol Salt Agar	Prepared by the media kitchen, Department of Biochemistry University of Oxford	N/A
LB medium	Prepared by the media kitchen, Department of Biochemistry University of Oxford	N/A
M9 Solution	Prepared by the media kitchen, Department of Biochemistry University of Oxford	recipe from wormbook: http://www.wormbook.org/chapters/www_strainmaintain/strainmaintain.html
NGM Agar	Prepared by the media kitchen, Department of Biochemistry University of Oxford	recipe from wormbook: http://www.wormbook.org/chapters/www_strainmaintain/strainmaintain.html

**Experimental models: Organisms/strains**		

Model Organism: *Caenorhabditis elegans strain* EVD00	H. Teotonio	EVD00
Model Organism: *Staphylococcus aureus* strain MSSA476	M. Horsburgh	MSSA476
Model Organism: *Enterococcus faecalis*	M. Horsburgh	OG1RF

**Software and algorithms**		

R version 4.0.2	https://www.r-project.org	N/A

### Resource availability

#### Lead contact

Further information and requests for resources and materials should be directed to and will be fulfilled by the lead contact, Kayla C. King (kayla.king@zoo.ox.ac.uk).

#### Materials availability

Selection lines are stored at −80°C at the University of Oxford and are available on request from Prof. Kayla King.

### Experimental model and subject details

Host selection lines were initiated from the *C. elegans* strain EVD00.^37^ Stocks were kept in buffer (20% DMSO) at –80°C. This population is dioecious and a representative mixture of male and female worms were used in all experiments and assays. Worms stocks were cultured on NGM plates seeded with non-pathogenic *Salmonella* food bacteria at 20°C.^53^ Parasite selection lines were initiated from *S. aureus* MSSA476. Stocks were kept in 25% glycerol at −80°C. The protective bacterium used in the experiment was *E. faecalis* OG1RF cultured under standard conditions (overnight culture in Todd-Hewitt (THB broth) from frozen stock) prior to each experimental host exposure. Throughout experiments and assays overnight, liquid cultures of *E. faecalis* and *S. aureus* were prepared using THB and incubated at 30°C overnight. Individual colonies were isolated via streaking out overnight cultures onto Tryptic Soy Broth (TSB) agar plates.

### Method details

#### Experimental evolution

For 15 host generations (∼105 days), nematodes were co-passaged with *S. aureus* MSSA476 parasites alone (unprotected) or after growing from L1 to adult on *E. faecalis* strain OG1RF (protected) following the methods of Rafaluk-Mohr et al.^51^ with the crushed worm suspension spread on parasite selective media (Mannitol Salt Agar, MSA). THB culture was grown from 100 parasite colonies at 30°C overnight. Briefly, worm eggs were surface sterilized with sodium hypochlorite and synchronized by shaking at 20°C overnight in M9 buffer. L1 worms were pipetted onto 9cm NGM plates seeded with either 600 μL overnight food culture (unprotected treatment) or mixed 300 μL food and 300 μL *E. faecalis* overnight cultures (protected treatment). Approximately 2000 synchronized L1 worms were added to each plate, plates dried, and incubated at 20°C for 48 h. *S. aureus* plates were simultaneously prepared by first growing up cultures in THB at 30°C overnight, then seeding 9cm TSB plates with 100 μL and growing bacteria up again at 30°C overnight. Following 48 h on either food bacteria alone (unprotected) or mixed food bacteria/*E. faecalis* (protected), worms were washed four times through filter tips^52^ to separate worms and bacteria. Washed worms were transferred to *S. aureus* plates on which they were incubated for a further 24 h at 25°C. Finally, worms were separated and surface washed again by filter tip washing and resuspended in M9 buffer. 10% of the resuspended population was crushed with a plastic pestle and streaked out on MSA. One-hundred colonies were picked and used to start the next parasite generation. The remainder of the worm suspension was pipetted onto food bacteria seeded NGM plates and allowed to lay eggs at 20°C for 48 h, these eggs were then surface sterilized and synchronized as described above and approximately 2000 L1 stage worms taken to start the generation. This cycle was repeated for 15 host generations.

#### Phenotypic assays

Survival assays were carried out as described in Rafaluk-Mohr et al.^51^ Briefly, evolved worm populations were thawed on food bacteria seeded plates and eggs surface sterilized with sodium hypochlorite and synchronized in M9 at 20°C overnight as described above. Survival assays were carried out on 6cm plates with approximated 200 worms per plate. Worms were grown on food seeded NGM plates for 48 h prior to parasite exposure for assays under unprotected conditions or 200 μL food bacteria plus 200 μL *E. faecalis* for assays under protected condition for 24 h. Dead and alive hosts were counted. Parasite load assays followed the same protocol, but ten *C. elegans* per replicate were picked, rinsed in M9 buffer and crushed to release gut bacteria then plated on MSA, incubated at 30°C for 48 h, and colony-forming units (CFUs) counted. For fecundity assays, the procedure was initially identical to that for survival assays, with the additional step that following parasite exposure, ten worms were picked onto NGM plates seeded with food bacteria. After 48 h, the number of eggs were counted.

#### Genome extraction

To obtain worm DNA, populations were grown on NGM plates with 600 μL food bacteria at 20°C. At population sizes of ∼3000 worms, plates were washed with M9 containing 0.1% Triton X. Worms were freeze-thawed repeatedly (dry ice to 60°C) to break the cuticles. DNA was extracted using QIAGEN blood and tissue kit. Library preparation and sequencing was performed by University of Liverpool CGR (BioProject: PRJNA673790). Parasite DNA was obtained following Ford et al.^16^ To obtain *S. aureus* DNA, 40 clones per sample from time points 0 (ancestral strain), 8, and 15 were grown overnight in 200 μL TSB at 30°C at 150 rpm. DNA was then extracted following the DNeasy Blood & Tissue Kit (QIAGEN) protocol. Bacterial clones were pooled together into 1.2 mL cultures and centrifuged at 8000 rpm for 3 min, where the pellet was re-suspended in 180 μL enzymatic lysis buffer (QIAGEN) and 4 μL RNase A (20 mg mL^-1^) (Sigma-Aldrich). After an incubation period at 37°C for 2 h, 25 μL protinase K (QIAGEN) and 200 μL Buffer AL (without ethanol) was added to samples. The solution was further incubated at 56°C for 30 min, before adding 200 μL ethanol and samples vortexed. DNA purification then followed the DNeasy Blood and Tissue Spin-column protocol (QIAGEN). Purity and quality of extracted DNA was assessed using the Nanodrop ND-100 and Qubit^â^ Fluorometer. Library preparation and whole-genome sequencing (HiSeq4000) performed by Oxford Genomics Centre (BioProject: PRJNA673790).

### Quantification and statistical analysis

Phenotypic data were analyzed using general linearized models with appropriate distributions (binomial or quasibinomial for survival data, depending on level of dispersion determined by diagnostic plots, Gaussian for CFU and fecundity data, determined by histograms). The significance threshold was defined as p < 0.05, where appropriate, p values were adjusted for multiple testing using the fdr method. The sample size n for each assay, defined as the total number of worm populations assayed (plates) can be found in the figure legends. Means and SEMs are represented in the figures.

Host genomic data were processed, and variants called using an in-house pipeline by the CGR Liverpool. To determine selection mode, GLMs were conducted for each genome position, for each treatment between the ancestral to middle experimental time-points, as well as the middle to end. Selection coefficients were calculated as in Papkou et al.^25^ Alleles that changed in frequency in the same direction in both time periods were classified as being under directional selection, whereas when the frequency changed in opposite directions they were classified as being under fluctuating selection. The relationships between recombination rate and number of alleles under selection were analyzed using a series of linear models. Parasite genomic data were processed and variants called following Ford et al.^16^ Phylogenetic trees for the parasite populations were calculated from Euclidean distances of variants as described in Ford et al.^16^ Detailed protocols are available upon request.

We calculated the Euclidean genetic distance of each *S. aureus* parasite population from the ancestor (Figure S2). Pairwise distances between treatments were significantly greater than distances from the ancestor (t = 2.6553, d.f. = 6.4692, p value = 0.03516). This result indicates significant divergence in parasites coevolving with unprotected and protected host populations. We performed a permutation anova comparing the distances of affected variants between treatments at passage 8 and 15 (P 8: F = 0.96, d.f. = 1,6, p = 0.812, P15: F = 0.99, d.f. = 1,6, p = 0.698), and distances of affected variants between those time-points for each treatment (protected: F = 0.78, d.f. = 1,6, p = 0.772, unprotected: F = 0.82, d.f. = 1,6, p = 1.0). We found that the treatments did not differ in the parasite loci that acquired mutations. A KEGG analysis showed no overall significant differences in gene groups acquiring mutations among treatments or time points (Table S2, Figures S3). Furthermore, although there was no significant difference in the number of alleles under each type of selection in the parasite (Chi-sq test), more alleles went to fixation in the unprotected treatment. A greater number of alleles underwent extreme changes in frequency (Figure S1), consistent with the higher levels of divergence seen in this treatment (Figure S2).

## Data Availability

• All sequencing data have been deposited at NCBI (BioProject: PRJNA673790) and are publicly available as of the date of publication. All phenotypic data have been published in Mendeley Data (https://doi.org/10.17632/8tw4cfhzbc.1) and are publicly available as of the date of publication. • R code used for the analyses in the paper is available on request. • Any additional information required to reanalyze the data reported in this paper is available from the lead contact upon request.

## Supplemental information

Document S1. Figures S1–S4 and Table S2

Table S1. Panther Slim GO terms for genes under selection in each treatment and for each mode of selection, related to Figure 4 A) Terms significantly under or over-represented in the unprotected treatment for i) alleles under arms race selection and ii) alleles under red queen selection. B) Terms significantly under or over-represented in the protected treatment for i) alleles under arms race selection and ii) alleles under red queen selection.

Table S3. Complete list of parasite variants, related to Figure 4

Document S2. Article plus supplemental information

## References

[1] Paterson S., Vogwill T., Buckling A., Benmayor R., Spiers A.J., Thomson N.R., Quail M., Smith F., Walker D., Libberton B. (2010). Antagonistic coevolution accelerates molecular evolution. Nature.

[2] Bell G. (2019).

[3] Hamilton W.D. (1980). Sex versus non-sex versus parasite. Oikos.

[4] Betts A., Rafaluk C., King K.C. (2016). Host and parasite evolution in a tangled bank. Trends Parasitol..

[5] Clay K. (2014). Defensive symbiosis: a microbial perspective. Funct. Ecol..

[6] Ford S.A., King K.C. (2016). Harnessing the power of defensive microbes: evolutionary implications in nature and disease control. PLoS Pathog..

[7] Vorburger C., Perlman S.J. (2018). The role of defensive symbionts in host-parasite coevolution. Biol. Rev. Camb. Philos. Soc..

[8] Heikkilä M.P., Saris P.E.J. (2003). Inhibition of Staphylococcus aureus by the commensal bacteria of human milk. J. Appl. Microbiol..

[9] Martín-Platero A.M., Valdivia E., Ruíz-Rodríguez M., Soler J.J., Martín-Vivaldi M., Maqueda M., Martínez-Bueno M. (2006). Characterization of antimicrobial substances produced by Enterococcus faecalis MRR 10-3, isolated from the uropygial gland of the hoopoe (Upupa epops). Appl. Environ. Microbiol..

[10] Martín-Vivaldi M., Peña A., Peralta-Sánchez J.M., Sánchez L., Ananou S., Ruiz-Rodríguez M., Soler J.J. (2010). Antimicrobial chemicals in hoopoe preen secretions are produced by symbiotic bacteria. Proc. Biol. Sci..

[11] Kommineni S., Bretl D.J., Lam V., Chakraborty R., Hayward M., Simpson P., Cao Y., Bousounis P., Kristich C.J., Salzman N.H. (2015). Bacteriocin production augments niche competition by enterococci in the mammalian gastrointestinal tract. Nature.

[12] Montalvo-Katz S., Huang H., Appel M.D., Berg M., Shapira M. (2013). Association with soil bacteria enhances p38-dependent infection resistance in Caenorhabditis elegans. Infect. Immun..

[13] Rossouw W., Korsten L. (2017). Cultivable microbiome of fresh white button mushrooms. Lett. Appl. Microbiol..

[14] Sifri C.D., Begun J., Ausubel F.M., Calderwood S.B. (2003). Caenorhabditis elegans as a model host for Staphylococcus aureus pathogenesis. Infect. Immun..

[15] King K.C., Brockhurst M.A., Vasieva O., Paterson S., Betts A., Ford S.A., Frost C.L., Horsburgh M.J., Haldenby S., Hurst G.D. (2016). Rapid evolution of microbe-mediated protection against pathogens in a worm host. ISME J..

[16] Ford S.A., Kao D., Williams D., King K.C. (2016). Microbe-mediated host defence drives the evolution of reduced pathogen virulence. Nat. Commun..

[17] Råberg L., Sim D., Read A.F. (2007). Disentangling genetic variation for resistance and tolerance to infectious diseases in animals. Science.

[18] Kutzer M.A.M., Armitage S.A.O. (2016). Maximising fitness in the face of parasites: a review of host tolerance. Zoology.

[19] Pike V.L., Ford S.A., King K.C., Rafaluk-Mohr C. (2019). Fecundity compensation is dependent on the generalized stress response in a nematode host. Ecol. Evol..

[20] Sorci G. (2013). Immunity, resistance and tolerance in bird-parasite interactions. Parasite Immunol..

[21] King K.C., Bonsall M.B. (2017). The evolutionary and coevolutionary consequences of defensive microbes for host-parasite interactions. BMC Evol. Biol..

[22] Gelino S., Chang J.T., Kumsta C., She X., Davis A., Nguyen C., Panowski S., Hansen M. (2016). Intestinal autophagy improves healthspan and longevity in C. elegans during dietary restriction. PLoS Genet..

[23] Buckling A., Rainey P.B. (2002). Antagonistic coevolution between a bacterium and a bacteriophage. Proc. Biol. Sci..

[24] Lively C.M. (1999). Migration, virulence, and the geographic mosaic of adaptation by parasites. Am. Nat..

[25] Papkou A., Guzella T., Yang W., Koepper S., Pees B., Schalkowski R., Barg M.-C., Rosenstiel P.C., Teotónio H., Schulenburg H. (2019). The genomic basis of Red Queen dynamics during rapid reciprocal host-pathogen coevolution. Proc. Natl. Acad. Sci. USA.

[26] Rockman M.V., Kruglyak L. (2009). Recombinational landscape and population genomics of Caenorhabditis elegans. PLoS Genet..

[27] Betts A., Gray C., Zelek M., MacLean R.C., King K.C. (2018). High parasite diversity accelerates host adaptation and diversification. Science.

[28] Masri L., Branca A., Sheppard A.E., Papkou A., Laehnemann D., Guenther P.S., Prahl S., Saebelfeld M., Hollensteiner J., Liesegang H. (2015). Host-pathogen coevolution: the selective advantage of Bacillus thuringiensis virulence and its cry toxin genes. PLoS Biol..

[29] Best A., White A., Boots M. (2008). Maintenance of host variation in tolerance to pathogens and parasites. Proc. Natl. Acad. Sci. USA.

[30] Miller M.R., White A., Boots M. (2006). The evolution of parasites in response to tolerance in their hosts: the good, the bad, and apparent commensalism. Evolution.

[31] Best A., White A., Boots M. (2014). The coevolutionary implications of host tolerance. Evolution.

[32] Svensson E.I., Råberg L. (2010). Resistance and tolerance in animal enemy-victim coevolution. Trends Ecol. Evol..

[33] Morran L.T., Schmidt O.G., Gelarden I.A., Parrish R.C., Lively C.M. (2011). Running with the Red Queen: host-parasite coevolution selects for biparental sex. Science.

[34] Fischer O., Schmid-Hempel P. (2005). Selection by parasites may increase host recombination frequency. Biol. Lett..

[35] Kerstes N.A., Bérénos C., Schmid-Hempel P., Wegner K.M. (2012). Antagonistic experimental coevolution with a parasite increases host recombination frequency. BMC Evol. Biol..

[36] Singh N.D., Criscoe D.R., Skolfield S., Kohl K.P., Keebaugh E.S., Schlenke T.A. (2015). EVOLUTION. Fruit flies diversify their offspring in response to parasite infection. Science.

[37] Theologidis I., Chelo I.M., Goy C., Teotónio H. (2014). Reproductive assurance drives transitions to self-fertilization in experimental Caenorhabditis elegans. BMC Biol..

[38] Salathé M., Kouyos R.D., Regoes R.R., Bonhoeffer S. (2008). Rapid parasite adaptation drives selection for high recombination rates. Evolution.

[39] Martinez J., Cogni R., Cao C., Smith S., Illingworth C.J.R., Jiggins F.M. (2016). Addicted? Reduced host resistance in populations with defensive symbionts. Proc. Biol. Sci..

[40] Koch H., Schmid-Hempel P. (2012). Gut microbiota instead of host genotype drive the specificity in the interaction of a natural host-parasite system. Ecol. Lett..

[41] Agrawal A., Lively C.M. (2002). Infection genetics: gene-for-gene versus matching-alleles models and all points in between. Evol. Ecol. Res..

[42] Best A., Ashby B., White A., Bowers R., Buckling A., Koskella B., Boots M. (2017). Host-parasite fluctuating selection in the absence of specificity. Proc. Biol. Sci..

[43] Brucker R.M., Bordenstein S.R. (2013). The hologenomic basis of speciation: gut bacteria cause hybrid lethality in the genus Nasonia. Science.

[44] Kolodny O., Callahan B.J., Douglas A.E. (2020). The role of the microbiome in host evolution. Philos. Trans. R. Soc. Lond. B Biol. Sci..

[45] Kolodny O., Schulenburg H. (2020). Microbiome-mediated plasticity directs host evolution along several distinct time scales. Philos. Trans. R. Soc. Lond. B Biol. Sci..

[46] Hawkes C.V., Bull J.J., Lau J.A. (2020). Symbiosis and stress: how plant microbiomes affect host evolution. Philos. Trans. R. Soc. Lond. B Biol. Sci..

[47] Metcalf C.J.E., Koskella B. (2019). Protective microbiomes can limit the evolution of host pathogen defense. Evol Lett.

[48] Hoang K.L., Gerardo N.M., Morran L.T. (2021). Association with a novel protective microbe facilitates host adaptation to a stressful environment. Evol. Lett..

[49] Råberg L., Graham A.L., Read A.F. (2009). Decomposing health: tolerance and resistance to parasites in animals. Philos. Trans. R. Soc. Lond. B Biol. Sci..

[50] Beaurepaire A.L., Moro A., Mondet F., Le Conte Y., Neumann P., Locke B. (2019). Population genetics of ectoparasitic mites suggest arms race with honeybee hosts. Sci. Rep..

[51] Rafaluk-Mohr C., Ashby B., Dahan D.A., King K.C. (2018). Mutual fitness benefits arise during coevolution in a nematode-defensive microbe model. Evol. Lett..

[52] Jansen G., Crummenerl L.L., Gilbert F., Mohr T., Pfefferkorn R., Thänert R., Rosenstiel P., Schulenburg H. (2015). Evolutionary transition from pathogenicity to commensalism: global regulator mutations mediate fitness gains through virulence attenuation. Mol. Biol. Evol..

[53] Kloock A., Bonsall M.B., King K.C. (2020). Evolution and maintenance of microbe-mediated protection under occasional pathogen infection. Ecol. Evol..

