# Supplementary material for: Microbial protection favors parasite tolerance and alters host-parasite coevolutionary dynamics
Source: Curr Biol. 2022 Apr 11;32(7):1593–1598.e3. doi: 10.1016/j.cub.2022.01.063 (PMC9355892; doi:10.1016/j.cub.2022.01.063)
Supplement: Document S1. Figures S1–S4 and Table S2 [file mmc1.pdf]

Current Biology, Volume *32*

Supplemental Information

Microbial protection favors parasite tolerance and alters host-parasite coevolutionary dynamics

Charlotte Rafaluk-Mohr, Michael Gerth, Jordan E. Sealey, Alice K.E. Ekroth, Aziz A. Aboobaker, Anke Kloock, and Kayla C. King

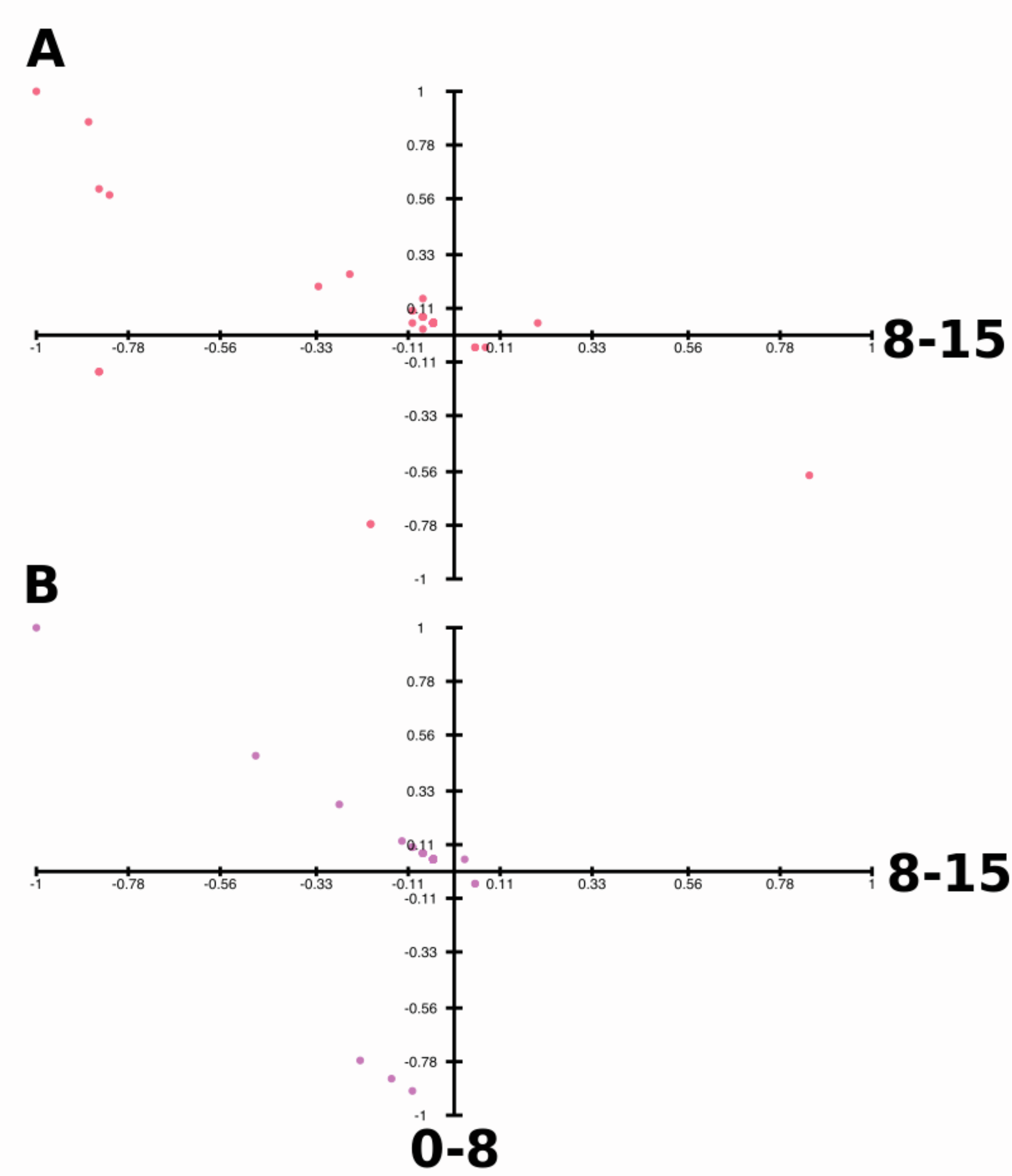

Figure S1. **Absolute change in variants in the parasite for each half of the evolution experiment. Related to Figure 4.** Changes in the same (positive-positive or negative-negative direction) indicate directional selection, whereas changes in opposing directions (positive-negative or negative-positive) suggest fluctuating selection.

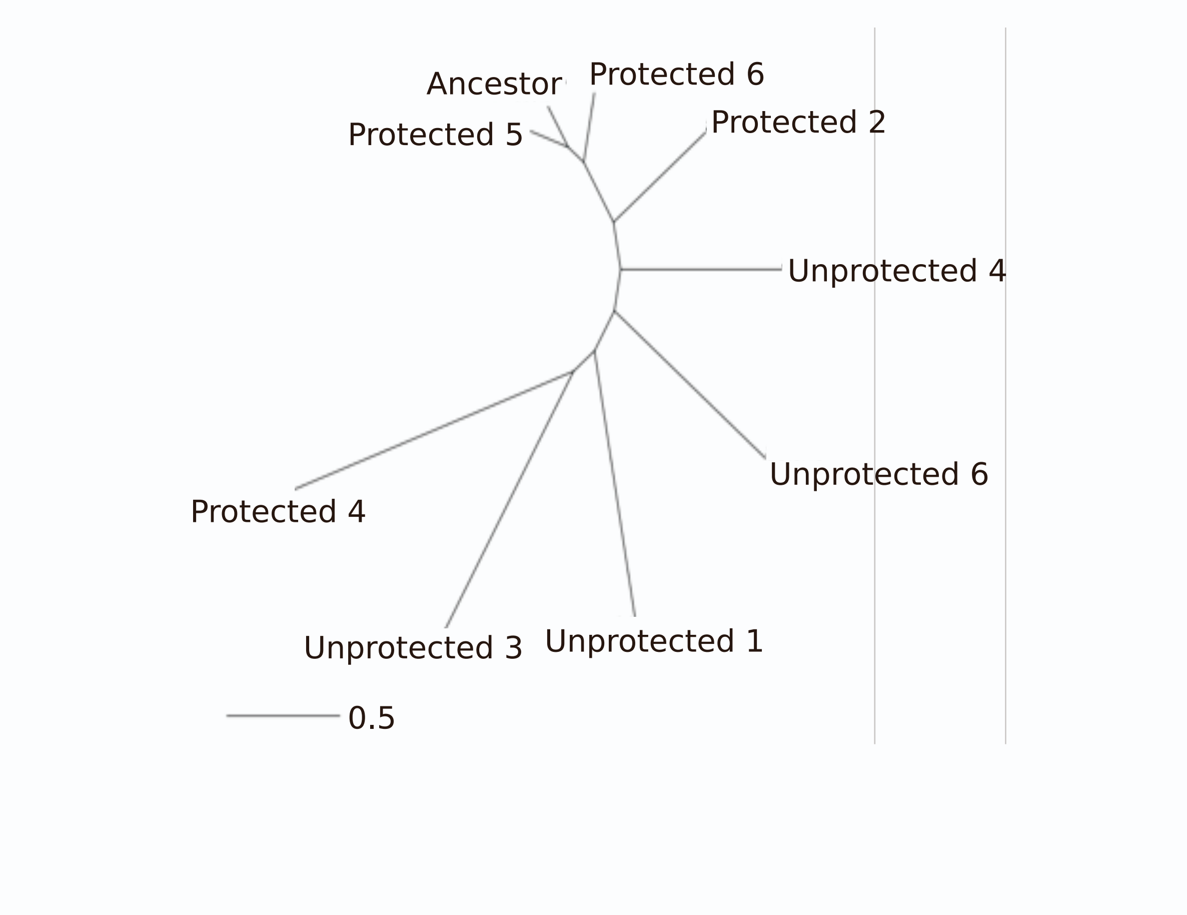

Figure S2. **Parasite genomic evolution during unprotected and protected coevolution. Related to Figure 4.** Tree showing the relationship between coevolved parasite populations and ancestor based on Euclidean genetic distances.

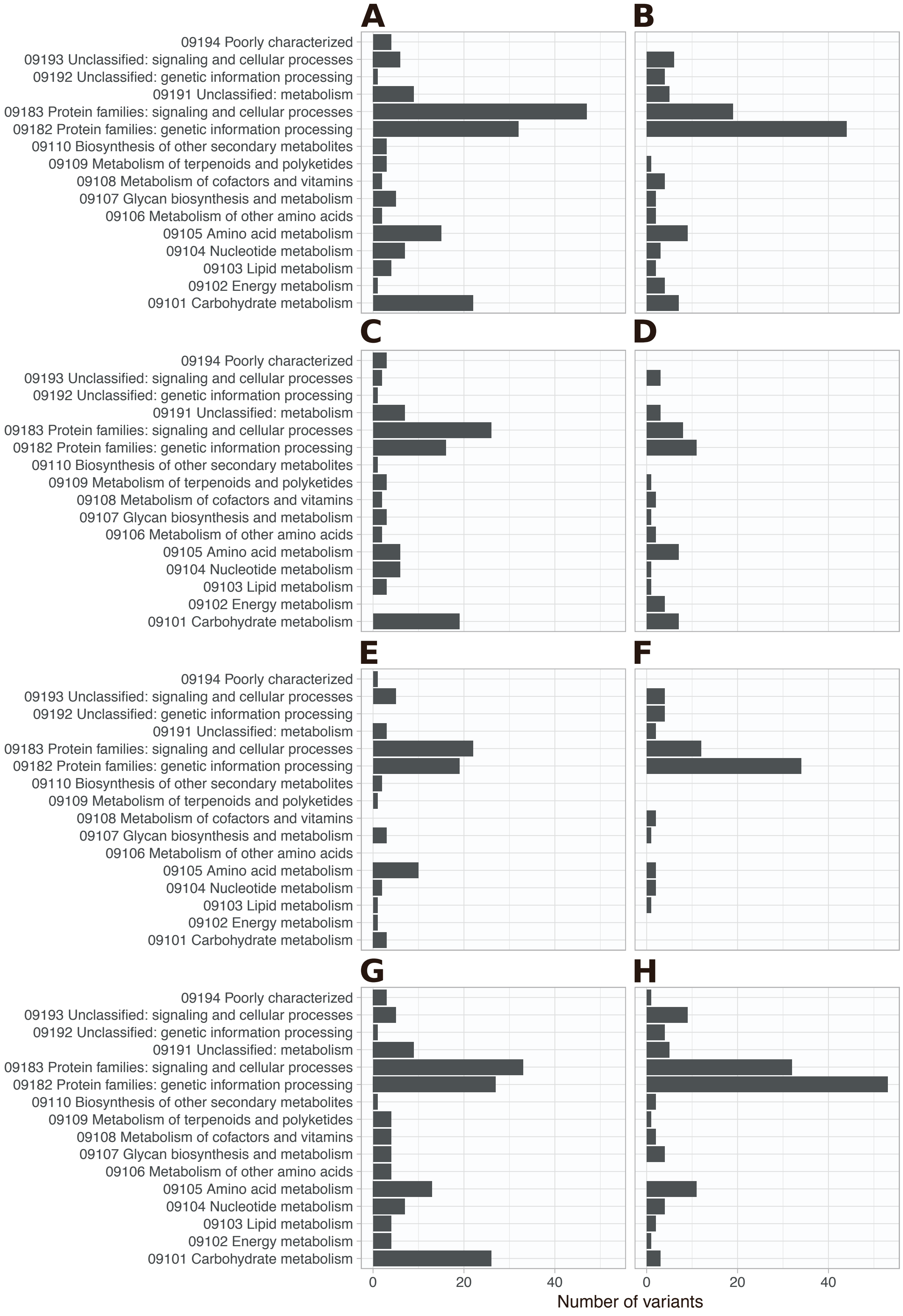

Figure S3. **Variant counts in each KEGG category. Related to Figure 4.** For A) the protected treatment overall; B) the unprotected treatment overall; C) the protected treatment at generation 8; D) the unprotected treatment at generation 8; E) the protected treatment at generation 15; F) the unprotected treatment at generation 15; G) all treatments at generation 8 and H) all treatments at generation 15.

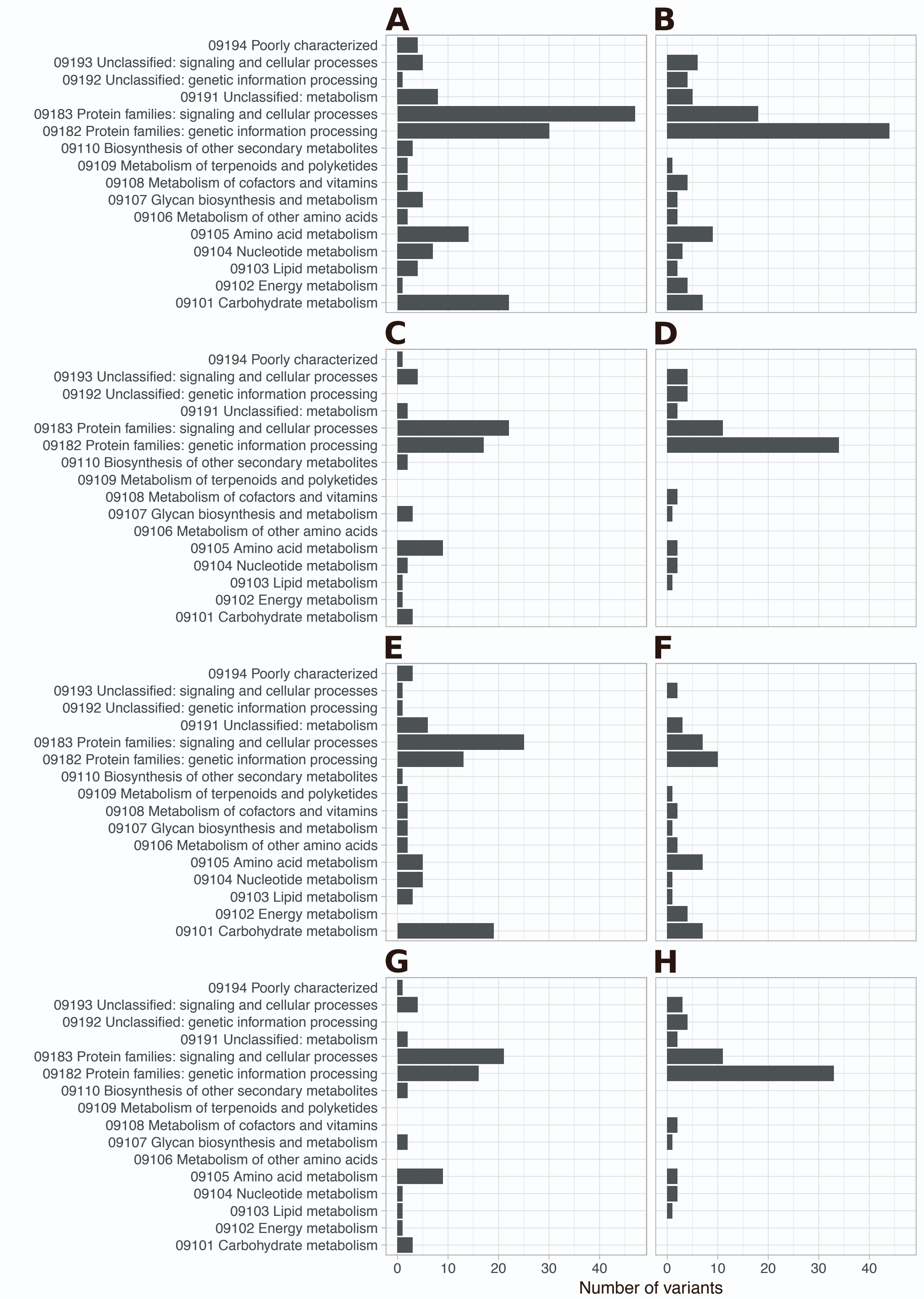

Figure S4. **Variant counts in each category Related to Figure 4.** For A) Alleles that changed in frequency between time points in the protected treatment; B) Alleles that changed in frequency between time points in the unprotected treatment; C) Alleles that increased in frequency at generation 15 in the protected treatment; D) Alleles that increased in frequency at generation 15 in the unprotected treatment; E) Alleles that decreased in frequency at generation 15 in the protected treatment; F) Alleles that decreased in frequency at generation 15 in the unprotected treatment; G) Loci at which new variants emerged in the protected treatment; H) Loci at which new variants emerged in the unprotected treatment.

Comparison group 1	Comparison group 2	P value
Protected both time points	Unprotected both time points	0.217
Protected generation 8	Unprotected generation 8	0.003
Protected generation 15	Unprotected generation 15	0.433
Protected generation 8	Protected generation 15	0.026
Unprotected generation 8	Unprotected generation 15	0.716
Generation 8 overall	Generation 15 overall	0.287
Protected treatment loci where variants changed in frequency between time points	Unprotected treatment loci where variants changed in frequency between time points	0.312
Protected treatment loci with increased frequency of variants at generation 15	Unprotected treatment loci with increased frequency of variants at generation 15	0.384
Protected treatment loci with decreased frequency of variants at generation 15	Unprotected treatment loci with decreased frequency of variants at generation 15	0.099
Protected treatment loci with new variants at generation 15	Unprotected treatment loci with new variants at generation 15	0.824

Table S2. Fisher exact tests of KEGG pathway comparisons. Related to Figure 4.
